# Comprehensive identification of lactate metabolism-related genes in recurrent spontaneous abortion: An observational study integrating bioinformatics and experimental validation

**DOI:** 10.1097/MD.0000000000051139

**Published:** 2026-10-30

**Authors:** Daili Tang, Lin Chen, Huiling Zheng, Zhi Huang, Ting Lai

**Affiliations:** a Department of Eugenics and Genetics, Guiyang Maternity and Child Health Hospital, Guiyang, Guizhou, China.

**Keywords:** key genes, lactate metabolism, machine learning, molecular regulatory network, recurrent spontaneous abortion

## Abstract

Recurrent spontaneous abortion (RSA) is a complex multifactorial disease. Lactate metabolism (LM) has been implicated in RSA pathogenesis, but the underlying molecular mechanisms remain unclear. This observational study aimed to identify key LM-related genes (LM-RGs) in RSA and explore their potential regulatory mechanisms and therapeutic targets. The study combined bioinformatics analysis with experimental validation. The training dataset GSE26787 and validation dataset GSE165004 were obtained from the Gene Expression Omnibus database. Differentially expressed genes were identified using the limma package. Candidate LM-RGs were obtained by intersecting differentially expressed genes with 262 LM-RGs from the Gene Set Enrichment Analysis database. Hub genes were identified through protein–protein interaction network analysis using the CytoHubba plugin (National Cheng Kung University). Key genes were further screened using Boruta and random forest machine learning algorithms, followed by expression validation in both Gene Expression Omnibus datasets. Reverse transcription–quantitative polymerase chain reaction was performed on 10 RSA and 10 control endometrial samples to validate key gene expression. Gene set enrichment analysis and gene set variation analysis were conducted to explore functional pathways. Regulatory networks involving microRNAs, long noncoding RNAs, and transcription factors were constructed, and potential therapeutic drugs were predicted using the Drug Signatures Database. *CALR* (upregulated) and *PIEZO1* (downregulated) were identified as key genes in RSA and were functionally linked to pathways such as cytokine signaling. Furthermore, we delineated a regulatory network governing these genes, implicating the long noncoding RNA X-inactive specific transcript and shared transcription factors, including MYC proto-oncogene, bHLH transcription factor and vitamin D receptor. We propose several candidate drugs, including estradiol, predicted to target both *CALR* and *PIEZO1*. This study identifies *CALR* and *PIEZO1* as key LM-RGs in RSA, with significant dysregulation confirmed in clinical samples. These genes are involved in cytokine signaling pathways and are regulated by complex molecular networks. The identification of common targeting drugs provides a foundation for developing novel therapeutic strategies for RSA. Further studies are warranted to validate these findings and explore their clinical applications.

## 1. Introduction

Miscarriage is the most common complication in early pregnancy, affecting at least 15% of diagnosed pregnancies and thus resulting in approximately 23 million miscarriages worldwide each year.^[1]^ Recurrent spontaneous abortion (RSA) refers to the occurrence of 2 or more consecutive pregnancy losses before 24 weeks of gestation. It is important to note that in clinical terminology, “abortion” typically refers to pregnancy loss before 24 weeks of gestation, as after 24 weeks, the fetus is considered potentially viable, and such an event is categorized as a preterm birth. RSA is a complex multifactorial disease affecting approximately 1% to 5% of women of reproductive age.^[2]^ This condition imposes significant psychological and physical burdens on many women trying to conceive.^[2]^ RSA is not only an early warning risk indicator for complications such as preterm labor and fetal growth restriction but also has a complex and varied pathogenesis involving chromosomal abnormalities and anatomical defects of the uterus.^[3]^ The effectiveness of current medical interventions for RSA is controversial, and although there has been progress in understanding the biological pathways of unexplained RSA, the exact molecular mechanisms are unknown.^[3,4]^

Lactic acid, as an important metabolic product, can be formed and utilized under fully aerobic conditions and can serve as an important regulator of systemic metabolic coordination.^[5]^ Changes in lactic acid levels may reflect the physiological response of the body when dealing with metabolic stress.^[6]^ Moreover, the accumulation of lactate in the tissue microenvironment is a characteristic of inflammatory diseases and cancer.^[6]^ Lactic acid is increasingly considered a signaling molecule.^[5]^ In patients with metabolic syndrome, increased fasting lactic acid levels are considered a marker of impaired metabolic health.^[7]^ Placental nutrient transport and metabolism are critical to fetal growth and development during pregnancy.^[8]^ By conducting metabolomics analysis on plasma samples from RSA groups and normal controls using gas and liquid chromatography–mass spectrometry, the lactate concentration in RSA patients was found to be significantly higher than that in the control group.^[9]^ Lactate metabolism (LM) was reported to play an important role in RSA mediated by decidual macrophages through the HIF-1α/SRC/LDHA pathway.^[10]^ It was also found that, in RSA, the decrease in succinate levels within cells could lead to the activation of the hydroxylase plant homeodomain, which in turn downregulated the hypoxia-inducible factor HIF signaling pathway.^[11]^ In addition, the plasma metabolite profiles of RSA patients show significant differences compared with those of the normal control group, particularly in changes related to LM, suggesting the role of LM in the pathogenesis of RSA.^[9]^ It is worth noting that in a mouse model prone to miscarriage, blocking lactate intake with AZD3965 (MCT-1 inhibitor) can rescue pregnancy, revealing the therapeutic potential of LM in RSA.^[10]^ However, the mechanism of action of LM in RSA remains unclear. Identifying key LM-related genes (LM-RGs) may offer novel clinical insights into RSA treatment and establish a theoretical basis for unraveling the mechanism underlying the relationship between RSA and LM.

Here, in this study, to facilitate understanding of the role of LM in RSA and reveal the molecular mechanisms of RSA, we aimed to identify key genes related to LM in RSA. Key genes were identified through multiple bioinformatics methods, including differential expression analysis, protein–protein interaction (PPI) analysis, and machine learning. A systematic analysis was then conducted based on these key genes using reverse transcription–quantitative polymerase chain reaction (RT-qPCR), gene set enrichment analysis (GSEA), gene set variation analysis (GSVA), molecular regulatory network analysis, and drug prediction analysis. We aimed to provide a new theoretical basis for revealing the mechanisms underlying the relationship between RSA and LM and to offer new ideas for the medical treatment of RSA.

## 2. Materials and methods

### 2.1. Data sources

We acquired all the datasets from the Gene Expression Omnibus database (https://www.ncbi.nlm.nih.gov/geo/). Specifically, the training set (GSE26787, platform: GPL570) included 5 normal endometrial tissue samples and 5 endometrial tissue samples from patients with RSA. The validation set (GSE165004, platform: GPL16699) included 24 normal endometrial tissue samples and 24 endometrial tissue samples from patients with RSA. Then, according to the literature,^[12]^ 262 LM-RGs were retrieved from the GSEA database (https://www.gsea-msigdb.org/gsea/index.jsp).

### 2.2. Differential expression analysis and identification of hub genes

Differentially expressed genes (DEGs) between RSA and control samples were identified in the GSE26787 dataset using the limma package (v3.54.1; R Foundation for Statistical Computing),^[13]^ with the criteria of |log_2_ fold change| > 0.5 and *P* < .05. The ggplot2 package (v3.3.6; R Foundation for Statistical Computing)^[14]^ and ComplexHeatmap package (v2.14.0; R Foundation for Statistical Computing)^[15]^ were employed to draw a volcano map and a heatmap. Notably, after sorting the log_2_FC values from largest to smallest, the top 10 upregulated and downregulated DEGs were labeled in the volcano plot, and their expression trends were visualized in the heatmap.

Afterwards, to acquire candidate genes related to LM, the ggvenn package (v0.1.9; R Foundation for Statistical Computing)^[16]^ was applied to obtain candidate genes from the intersection of DEGs and LM-RGs. Then, to further understand the pathways involved in the candidate genes, the clusterProfiler package (v4.7.1.003; R Foundation for Statistical Computing)^[17]^ was applied to conduct Gene Ontology (GO) and Kyoto Encyclopedia of Genes and Genomes (KEGG) analyses of the candidate genes (*P* < .05). GO included 3 parts, namely, biological processes (BPs), cellular components (CCs), and molecular functions (MFs). The top 5 entries for each section of GO were visualized, and the top 10 KEGG pathways were visualized by sorting the count values from largest to smallest.

Furthermore, to investigate whether there were interactions between candidate genes at the protein level, the candidate genes were imported into the Search Tool for the Retrieval of Interacting Genes/Proteins database (https://string-db.org/), and the PPI network was constructed (confidence > 0.15). After removal of isolated proteins, the network was visualized using Cytoscape (v3.9.1; National Resource for Network Biology).^[18]^ Finally, the Betweenness and BottleNeck algorithms in the CytoHubba plugin were employed to evaluate the importance of each node. The top 20 highest-rated nodes from each algorithm were selected, and their intersection was used to identify the hub genes. The ggvenn package (v0.1.9) was adopted to visualize the results.

### 2.3. Identification of key genes according to machine learning and expression verification

To further obtain key genes associated with LM in RSA, the Boruta package (v8.0.0; Miron Bartosz Kursa)^[19]^ was applied to obtain the final decisions (confirmed Boruta-feature genes). The Random Forest (RF) package (v4.7.1.1; R Foundation for Statistical Computing)^[20]^ was used to perform RF analysis on candidate genes, and feature genes were obtained based on whether the importance value was greater than the median. Next, the ggvenn package (v0.1.9) was applied to obtain common feature genes from the intersection of Boruta-feature genes and RF-featured genes. Ultimately, to identify key genes, the expression levels of feature genes in GSE26787 and GSE165004 were evaluated, and the Wilcoxon test was employed to evaluate the differences between RSA and control samples. The ggplot2 package (v3.3.6) was then applied to visualize the results. Notably, key genes were defined as feature genes that differed between RSA and control samples and had consistent trends in both datasets (*P* < .05).

### 2.4. RT-qPCR

To confirm the consistency of key gene expression with the bioinformatics results, key gene expression was verified by RT-qPCR. Ten normal endometrial tissues and 10 endometrial tissue samples from patients with RSA were collected, respectively. The normal endometrial tissues were obtained from patients undergoing induced abortion at 6 to 8 weeks of normal gestation, with villous tissues avoided during sampling. All these patients had no history of RSA, endocrine disorders, or uterine structural abnormalities. Endometrial tissues from RSA patients were collected from patients undergoing curettage due to embryonic arrest at 7 to 10 weeks of gestation, all of whom had experienced 2 or more spontaneous abortions. Villous tissues were also excluded to ensure pure endometrial stroma and glands. Immediately after collection, the samples were rinsed with normal saline to remove blood clots and debris, trimmed into small pieces of approximately 0.5 cm^3^, and then stored in a −80°C ultra-low-temperature freezer for subsequent experiments. All samples were transported on dry ice to prevent the degradation of RNA and proteins. It is important to note that all samples were collected from Guiyang Maternal and Child Health Care Hospital, Guiyang, Guizhou, China, with patients providing informed consent. This study was approved by the Ethics Committee of Guiyang Maternal and Child Health Care Hospital, Guiyang, Guizhou, China (approval number: 2025-23). Total RNA was extracted from normal samples and RSA endometrial tissue samples using TRIzol reagent (Ambion), and the RNA quality was evaluated using a spectrophotometer. Subsequently, cDNA synthesis was performed using the Master Mix of the SweScript First Strand cDNA synthesis kit (Thermo Fisher) for subsequent qPCR reactions. The reaction mixture contained 4 μL of 5× Reaction Buffer, 1 µL of SweScript RT I Enzyme Mix, 1 µL of Primer, 2 μg of total RNA, and nuclease-free water, with a total volume of 20 μL. The reaction conditions were as follows: incubation at 25°C for 5 minutes, continuous incubation at 25°C for 15 minutes, incubation at 85°C for 5 seconds, and then storage at 4°C. The qPCR reaction was carried out according to the manufacturer’s instructions. The qPCR reaction mixture included 3 μL of cDNA, 5 μL of 2× Universal Blue SYBR Green qPCR Master Mix, and 1 μL of each 10 μM forward and reverse primer. The reaction conditions were as follows: predenaturation at 95°C for 1 minute, denaturation at 95°C for 20 seconds, annealing at 60°C for 20 seconds, and amplification at 72°C for 30 seconds for 40 cycles. Each sample was tested in 3 replicates, and 2 technical replicates were set. The specificity of the primers was verified by melting curve analysis, and the results showed that all primers only showed 1 peak. Glyceraldehyde-3-phosphate dehydrogenase was used as the internal reference gene, and the relative gene expression level was calculated using the 2^−ΔΔCt^ method. The ΔCt value was the difference between the Ct value of the target gene and the Ct value of the internal reference gene, and the ΔΔCt value was the difference in ΔCt values between the RSA group and the control group. The primer sequences are shown in Table S1, Supplemental Digital Content 1. Statistical analysis and visualization of the data were performed using GraphPad Prism (v8.0; GraphPad Software, Inc.),^[21]^ and all experimental data were presented as mean ± standard deviation. The difference between the 2 groups was assessed by *t* test (*P* < .05).

### 2.5. GSEA and GSVA

To further understand the potential functions of the key genes, according to the GSE26787 dataset, the psych package (v2.2.9; R Foundation for Statistical Computing)^[22]^ was applied to analyze the associations between each key gene and other genes, and the list of genes was generated by sorting the correlations from largest to smallest. Afterwards, the background gene set “c2.kegg.v7.4.symbols.gmt” was obtained from the Molecular Signatures Database (https://www.gsea-msigdb.org/gsea/msigdb), and the clusterProfiler package (v4.7.1.003) was applied to conduct GSEA (|normalized enrichment score| > 1, *P* < .05). The top 5 marked pathways were visualized using the enrichplot package (v1.18.0; R Foundation for Statistical Computing).^[23]^ Besides, to compare the differences in enriched pathways between RSA and control samples, “c2.cp.kegg.v2023.1.Hs.symbols.gmt” was obtained from the Molecular Signatures Database as the background gene set. Subsequently, based on the expression matrices of the key genes in the GSE26787 dataset, the GSVA package (v1.46.0; Bioconductor Project, Universitat Pompeu Fabra)^[24]^ was applied to calculate the scores of individual key genes for the pathways in each sample. The limma package (v3.54.1) was utilized to analyze markedly enriched pathway variations between the RSA and control groups (*P* < .05, |*t*| > 2), and the top 10 markedly enriched pathways for upregulation and downregulation were visualized.

### 2.6. Gene–gene interaction (GGI) network and chromosome localization analysis

To identify genes associated with the functions of key genes, GeneMANIA (http://genemania.org/) was applied to predict genes associated with and involved in the functions of key genes and to visualize their interaction networks. Furthermore, the RCircos package (v1.2.2; National Cancer Institute, National Institutes of Health)^[25]^ was employed to analyze the distribution of key genes on chromosomes, providing a basis for subsequent applications of key genes.

### 2.7. Construction of regulatory networks

To investigate the possible expression regulation patterns of key genes, the MicroRNA Target Prediction Database (https://mirdb.org/) was applied to predict microRNAs (miRNAs) targeting key genes. Subsequently, the Encyclopedia of RNA Interactomes (StarBase) database (https://rnasysu.com/encori/) was applied to predict the long noncoding RNAs (lncRNAs) of key genes. Moreover, to investigate the transcription factors (TFs) that interact with key genes, NetworkAnalyst (https://www.networkanalyst.ca/NetworkAnalyst/) was used to predict the TFs targeting these genes. Finally, to investigate potential drugs for the treatment of RSA, the Drug Signatures Database (https://dsigdb.tanlab.org/) was employed to identify key genes for potential drugs, and the top 10 drugs were selected as potential therapeutics for RSA according to the interaction scores. The above interaction networks were visualized using Cytoscape software (v3.9.1).

### 2.8. Statistical analysis

R software (v4.2.2; R Foundation for Statistical Computing) was used to conduct bioinformatics analyses. The Wilcoxon test and *t* test were utilized to evaluate paired-sample comparisons. A *P* value < .05 was considered statistically significant.

## 3. Results

### 3.1. Candidate genes involved in pathways significantly associated with LM

A total of 3504 DEGs were identified between RSA and control samples; among these, 2708 were upregulated DEGs and 796 were downregulated DEGs in the RSA samples. The expression patterns of DEGs between groups exhibited distinct profiles (Fig. 1A and B). Subsequently, a total of 40 candidate genes were retained from the intersection of DEGs and LM-RGs (Fig. 1C). Moreover, the candidate genes were markedly enriched in 453 GO entries (*P* < .05). Among them, BP, CC, and MF were markedly enriched in 335, 46, and 72 entries, respectively (Table S2, Supplemental Digital Content 2). Specifically, there was marked enrichment mainly in mitochondrial gene expression (BP), mitochondrial inner membrane (CC), and aminoacyl-tRNA ligase activity (MF; Fig. 1D). Meanwhile, candidate genes were markedly enriched in 12 KEGG pathways (*P* < .05), including aminoacyl-tRNA biosynthesis, carbon metabolism, and pyruvate metabolism (Fig. 1E; Table S3, Supplemental Digital Content 3). These pathways were markedly associated with lactate and its metabolism, revealing the relevance of candidate genes to LM. Furthermore, there were 39 candidate genes encoding proteins that interacted with each other in the PPI network, with 255 interaction pairs identified. Among them, DARS2, SLC25A4, AARS2, SUF1, and YARS2 interacted with many proteins (Fig. 1F). Finally, by taking the intersection of the top 10 genes identified by the Betweenness and BottleNeck algorithms, a total of 16 hub genes were identified for subsequent analysis (Fig. 1G).

**Figure 1.**
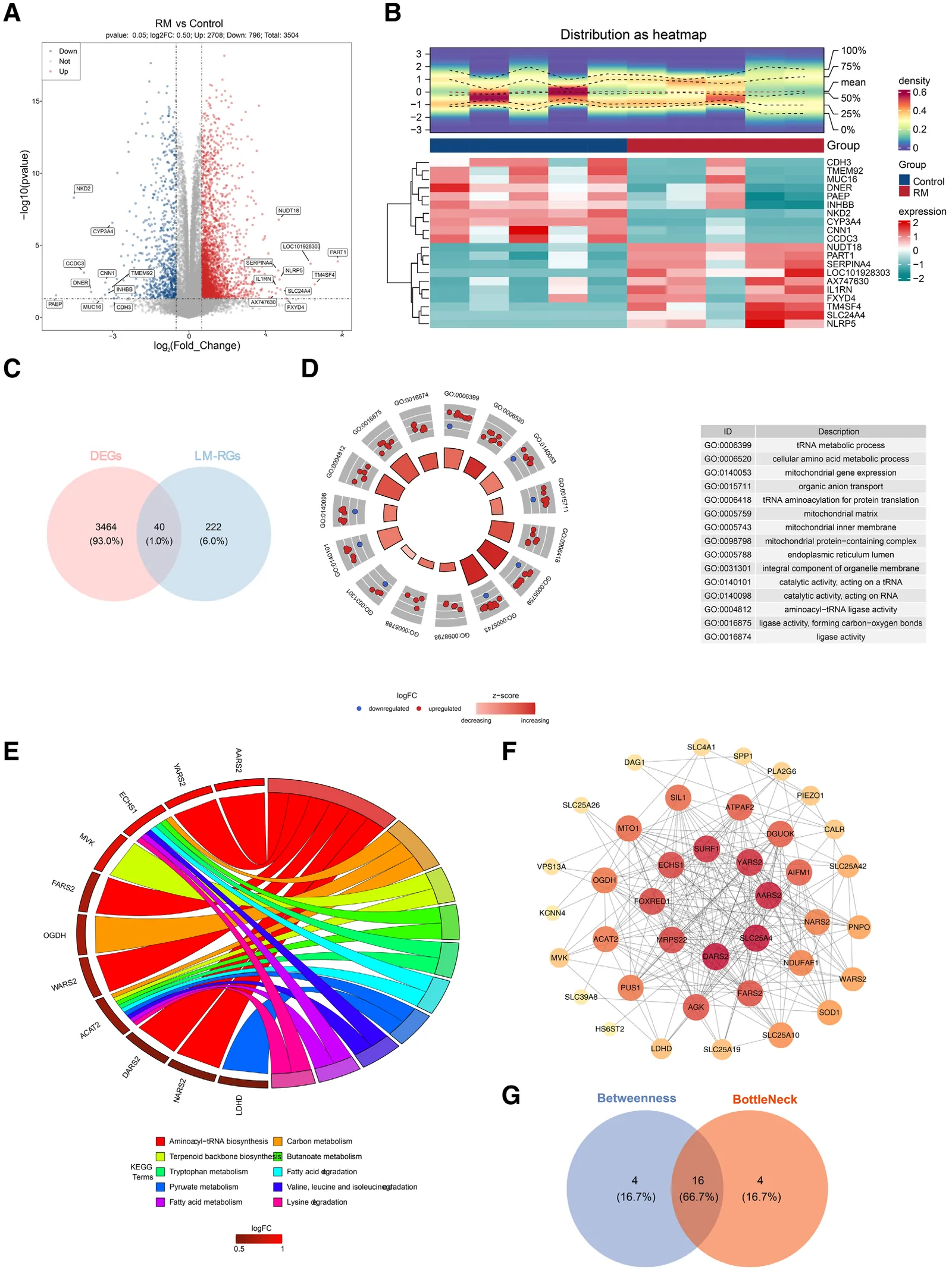
Identification of candidate genes related to lactate metabolism in RSA. (A) Volcano plot displaying differentially expressed genes (DEGs) between RSA and control samples. (B) Heatmap showing expression patterns of the top 20 DEGs. (C) Venn diagram illustrating the overlap between DEGs and lactate metabolism-related genes. (D) GO enrichment analysis of candidate genes. (E) KEGG pathway enrichment analysis. (F) PPI network of candidate genes. (G) Intersection of hub genes identified by the Betweenness and BottleNeck algorithms. GO = Gene Ontology, KEGG = Kyoto Encyclopedia of Genes and Genomes, PPI = protein–protein interaction, RM = recurrent miscarriage; RSA = recurrent spontaneous abortion, tRNA = transfer ribonucleic acid.

### 3.2. *CALR* and *PIEZO1* were key genes associated with LM in RSA

The 10 Boruta-feature genes were confirmed, namely, AARS2, *PIEZO1*, ECHS1, DARS2, SURF1, PUS1, SLC4A1, NDUFAF1, FARS2, and *CALR* (Fig. 2A). Meanwhile, in the RF analysis, 8 RF-feature genes were identified, namely, ECHS1, *CALR*, AIFM1, NDUFAF1, FARS2, AARS2, *PIEZO1*, and DARS2, which had importance values greater than the median (MeanDecreaseGini > 0.2940476; Fig. 2B). Subsequently, a total of 7 common feature genes were acquired (AARS2, *PIEZO1*, ECHS1, DARS2, NDUFAF1, FARS2, and *CALR*; Fig. 2C). Finally, according to the expression of the 7 feature genes in GSE26787 and GSE165004, we found that *CALR* and *PIEZO1* were significantly different between groups, and the trends were consistent in both datasets. Specifically, *CALR* expression was markedly upregulated in RSA samples, whereas *PIEZO1* expression was markedly downregulated (*P* < .05; Fig. 2D and E), thus identifying the key genes associated with LM in RSA. Moreover, expression analysis of clinical samples further validated the reliability of the bioinformatics analysis results. Specifically, compared with control samples, *CALR (P* < .01) and *PIEZO1 (P* < .05) expression was upregulated and downregulated, respectively, in RSA samples (Fig. 2F and G), consistent with the expression trends in GSE26787 and GSE165004.

**Figure 2.**
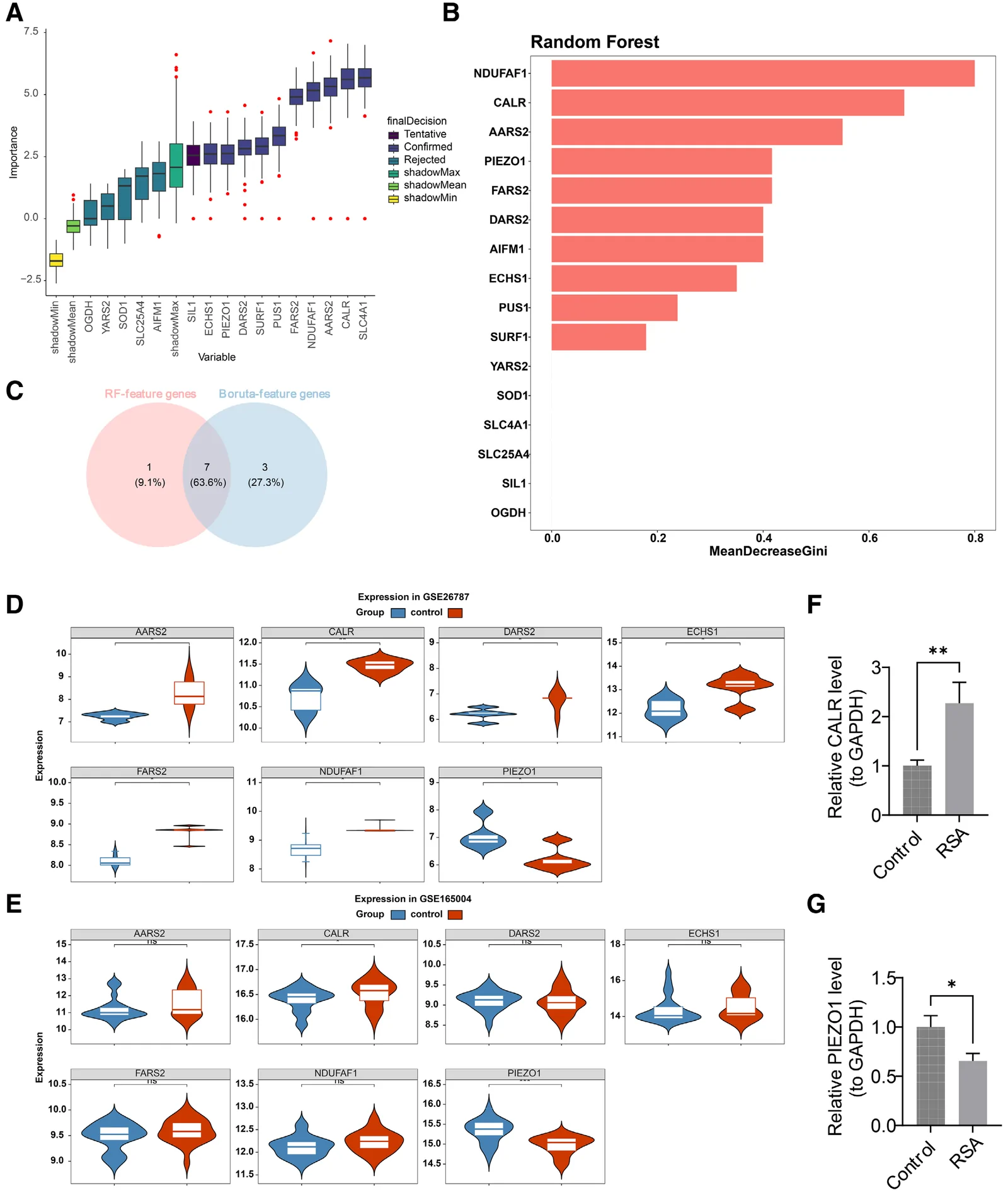
Screening and validation of key genes associated with RSA. (A) Boruta algorithm-selected feature genes. (B) Random forest analysis highlighting important genes. (C) Venn diagram of shared feature genes. (D, E) Expression validation of *CALR* and *PIEZO1* in GEO datasets. (F, G) RT-qPCR confirmation of *CALR* and *PIEZO1* expression in clinical samples. n = 10 for the RSA group and n = 10 for the control group; each sample was tested in 3 replicates, and 2 technical replicates were set. Data are presented as mean ± standard deviation. GAPDH = glyceraldehyde-3-phosphate dehydrogenase, GEO = Gene Expression Omnibus, RF = random forest, RSA = recurrent spontaneous abortion, RT-qPCR = reverse transcription–quantitative polymerase chain reaction.

### 3.3. Targeting these key pathways might offer novel ideas for RSA treatment

Next, we further investigated the ways in which key genes may be involved in RSA development through GSEA. The data revealed that *CALR* was markedly enriched in 28 pathways in total, with major involvement in oxidative phosphorylation, neuroactive ligand–receptor interaction, and cytokine–cytokine receptor interaction (Fig. 3A; Table S4, Supplemental Digital Content 4). *PIEZO1* was markedly enriched in 40 pathways, mainly involved in ribosome, natural killer cell-mediated cytotoxicity, and peroxisome (*P* < .05, |normalized enrichment score| > 1; Fig. 3B, Table S5, Supplemental Digital Content 5). Notably, the key genes were co-enriched in cytokine–cytokine receptor interaction, suggesting a synergistic role of key genes in RSA development. Then, 36 pathways were markedly enriched in the GSVA, of which 22 were upregulated in the RSA group (*P* < .05, |*t*-value| > 2; Table S6, Supplemental Digital Content 6). Specifically, the pathways basal TFs, riboflavin metabolism, spliceosome, and proteasome were activated in RSA, whereas primary immunodeficiency, cytokine–cytokine receptor interaction, and histidine metabolism were inhibited in RSA (Fig. 3C). This suggested that these pathways might be markedly associated with the progression of RSA and that targeting these pathways might provide new ideas for the treatment of RSA.

**Figure 3.**
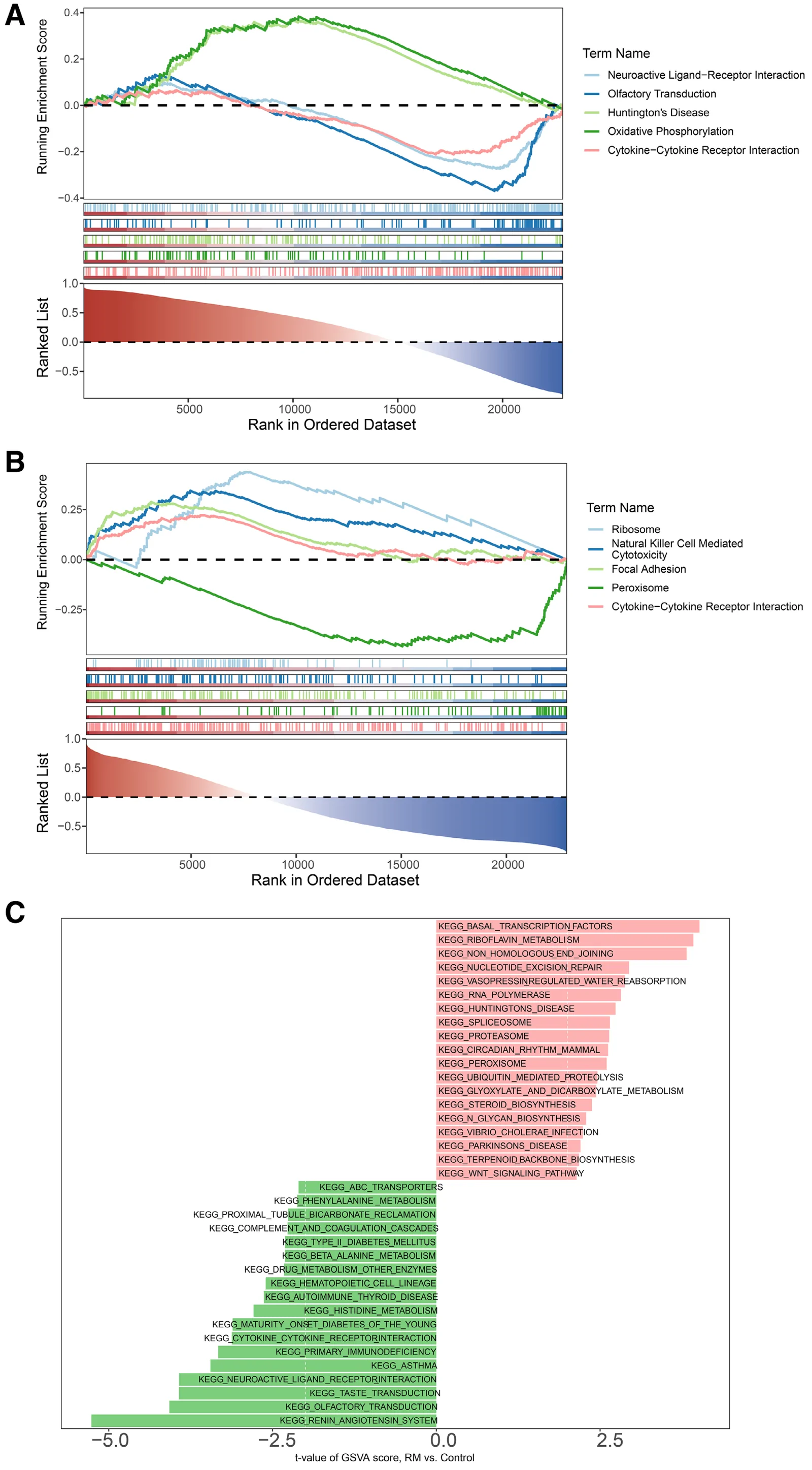
Functional enrichment analysis of key genes. (A, B) GSEA results for *CALR* and *PIEZO1*, showing enriched pathways. (C) GSVA heatmap depicting differentially activated pathways in RSA versus control samples. GSEA = gene set enrichment analysis, GSVA = gene set variation analysis, KEGG = Kyoto Encyclopedia of Genes and Genomes, RSA = recurrent spontaneous abortion.

### 3.4. GGI and chromosomal localization of *CALR* and *PIEZO1* were revealed

Moreover, the GGI demonstrated that the modes of interaction among *CALR*, *PIEZO1*, and 20 other relevant genes were mainly physical interactions, co-expression, and predicted interactions, and that the key genes might be involved in pathways mainly including integral component of the endoplasmic reticulum membrane, antigen processing and presentation of exogenous peptide antigen via major histocompatibility complex class I, and antigen processing and presentation of peptide antigen via major histocompatibility complex class I (Fig. 4A). Overall, understanding the patterns in which key genes interacted and the pathways involved helped to understand the ways in which key genes function, which in turn provided ideas for targeting these pathways to treat RSA.

**Figure 4.**
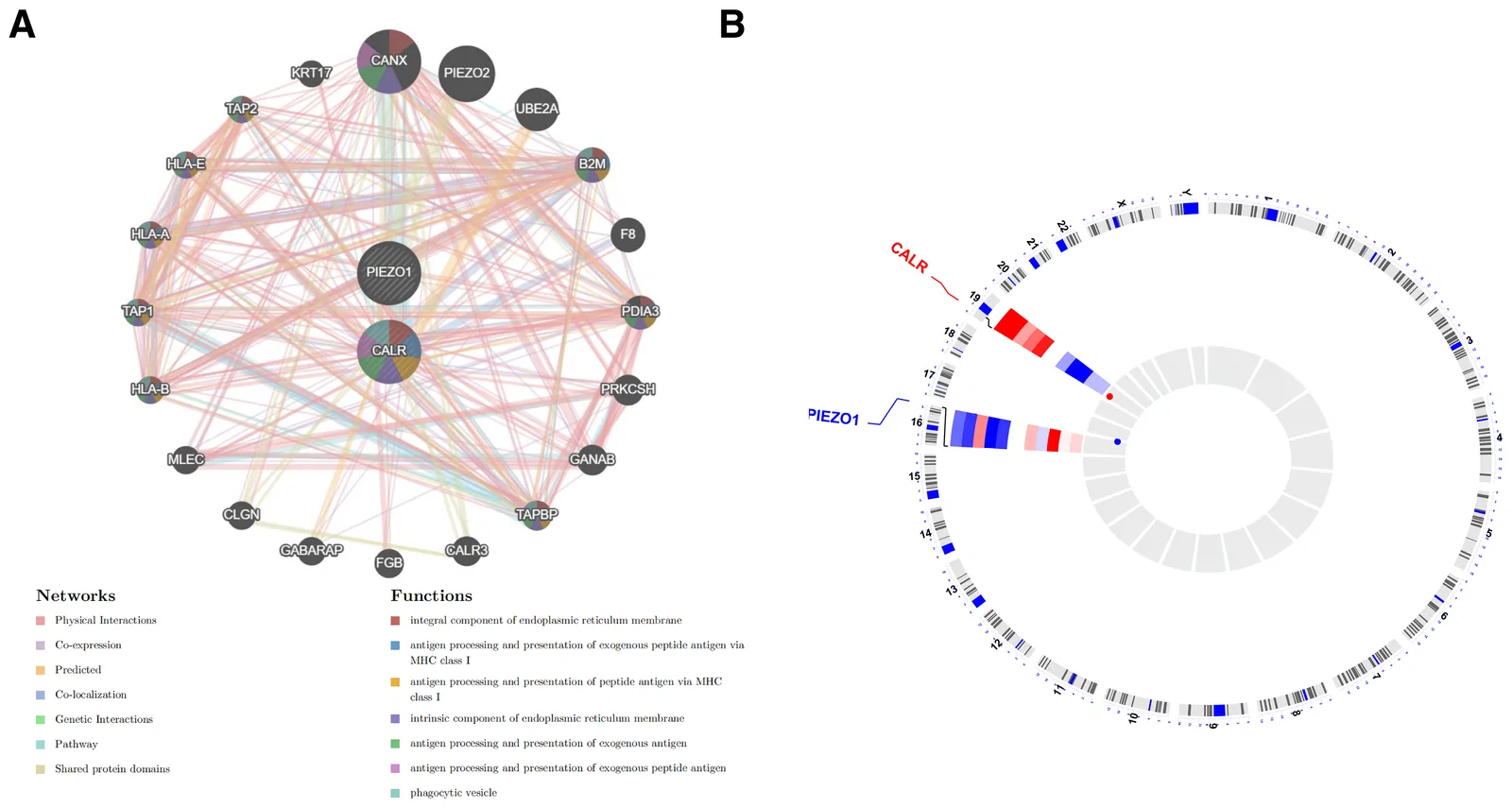
Interaction and chromosomal localization of key genes. (A) Gene–gene interaction network of *CALR* and *PIEZO1* with associated genes. (B) Chromosomal distribution of *CALR* (chr19) and *PIEZO1* (chr16). Red: *CALR*; Blue: *PIEZO1*.

Furthermore, *CALR* and *PIEZO1* were localized to chromosomes 19 and 16, respectively (Fig. 4B). Clarifying the specific locations of key genes on chromosomes helped provide insight into the BPs and signaling pathways in which key genes might be involved, providing important clues for understanding the genetic basis and molecular mechanisms of RSA.

### 3.5. Complex regulatory network of *CALR* and *PIEZO1* was revealed

*CALR* and *PIEZO1* were predicted to have 3 (hsa-miR-31-5p, hsa-miR-1913, and hsa-miR-4731-5p) and 2 (hsa-miR-101-3p and hsa-miR-139-5p) miRNAs, respectively. Based on this, 40 lncRNAs were identified for the 5 miRNAs. Notably, NEAT1 was co-predicted by 5 miRNAs, and X-inactive specific transcript was co-predicted by miRNAs other than hsa-miR-1913, revealing the possibility that they could co-regulate the 2 key genes (Fig. 5A). Subsequently, *CALR* and *PIEZO1* were predicted to interact with 44 and 24 TFs, respectively, of which 9 TFs, such as MYC proto-oncogene, bHLH transcription factor, vitamin D receptor, and TP53, were common to both (Fig. 5B). Overall, *CALR* possessed a relatively more complex regulatory network than *PIEZO1*, which provided a theoretical basis for targeting and modulating *CALR* and *PIEZO1* for the treatment of RSA.

**Figure 5.**
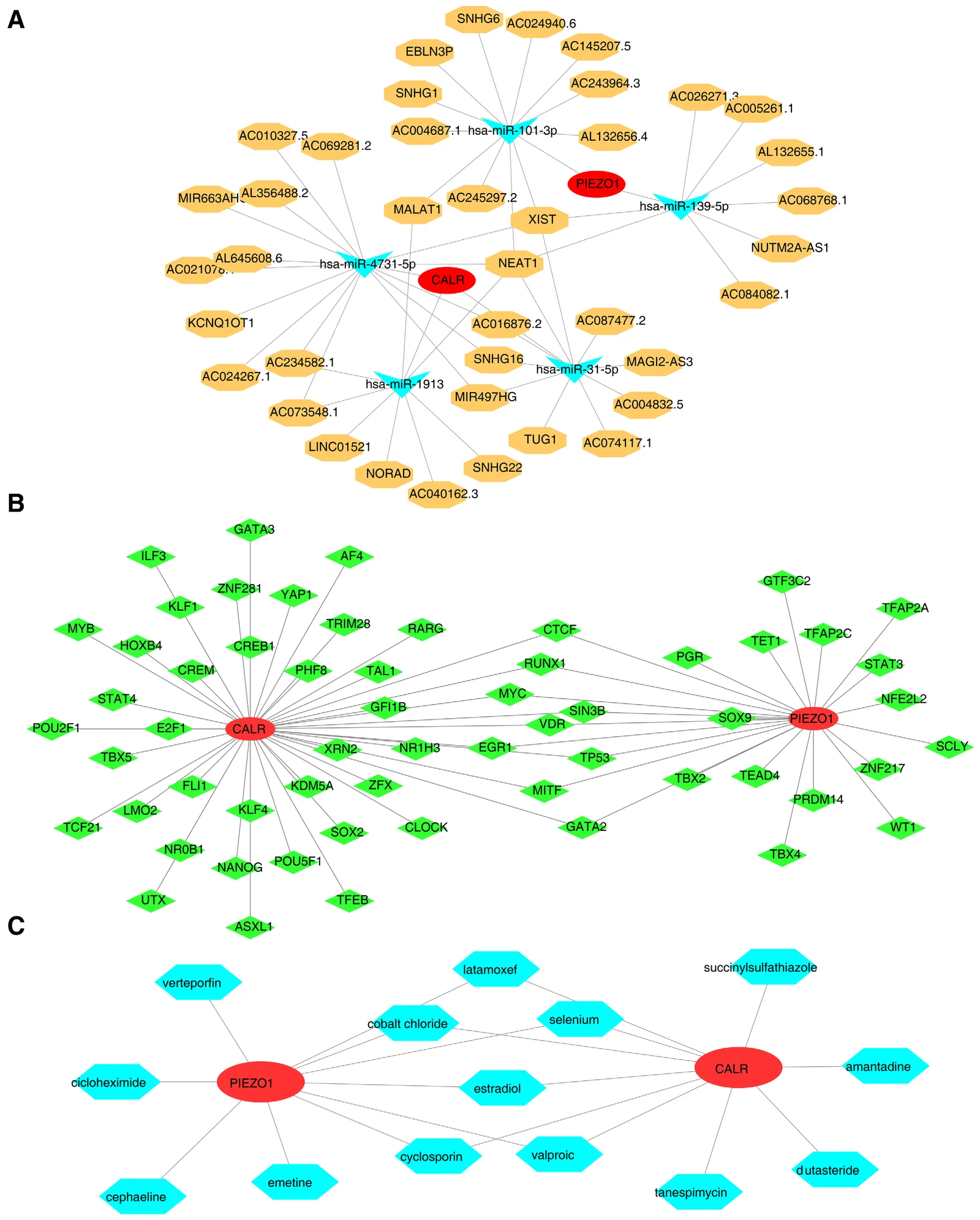
Regulatory networks and drug prediction for key genes. (A) miRNA–lncRNA co-regulation network targeting *CALR* and *PIEZO1*. (B) Shared transcription factors (TFs) regulating both genes. (C) Potential therapeutic drugs targeting *CALR* and *PIEZO1*. lncRNAs = long noncoding RNAs, miRNAs = microRNAs.

Moreover, the construction of drug regulatory networks further revealed the clinical potential of targeting key genes to treat RSA. Specifically, *CALR* and *PIEZO1* were predicted to have 77 and 13 targeting drugs, respectively. The top 10 targeting drugs based on the interaction scores contained 6 drugs in common, namely, selenium, latamoxef, cobalt chloride, estradiol, cyclosporin, and valproic acid (Fig. 5C). It is crucial to note that this is a bioinformatics-based prediction, and these findings are for exploratory purposes only. For instance, valproic acid, while identified in our analysis, is contraindicated in pregnancy due to its well-documented teratogenic effects. Therefore, any potential therapeutic application would require extensive preclinical and clinical evaluation to assess safety and efficacy and must strictly adhere to clinical guidelines and contraindications. In short, the availability of these drugs was expected to enable the treatment of RSA by targeting key genes.

## 4. Discussion

RSA is a multifactorial disease, and the study of metabolomics has provided a new perspective for revealing the potential mechanisms of RSA.^[26]^ Elucidating the role of LM in RSA indeed facilitates a better understanding of its pathogenesis and provides guidance for its clinical treatment.^[27]^ Here, by performing bioinformatics analyses and systemic validation, we identified *CALR* and *PIEZO1* as key LM-RGs in RSA, which were significantly upregulated and downregulated in RSA, respectively. The 2 key genes were involved in important pathways such as cytokine–cytokine receptor interaction. By constructing molecular regulatory networks, *CALR* was predicted to have a more complex regulatory network than *PIEZO1*. *CALR* and *PIEZO1* were co-regulated by lncRNAs and TFs. Ultimately, *CALR* and *PIEZO1* had 6 common targeting agents, such as estradiol and cyclosporin, which provided a basis for targeting key genes to treat RSA.

During the process of embryo nidation, syncytiotrophoblasts depend on a large amount of energy produced by glycolysis for rapid growth and penetration.^[28]^ Metabolic disorders in trophoblast cells can lead to RSA.^[28]^ Particularly, lactate resulting from glycolysis within trophoblast cells during early pregnancy can drive the polarization of M2 macrophages and maintain an anti-inflammatory state at the maternal-fetal interface.^[29]^ However, in the villi of RSA patients, there is an abnormal increase in the copper content of the aldehyde oxidase pseudogene. This increase hindered the polarization of M2 macrophages.^[29]^ Besides, metabolomics analysis of the uterine fluid from RSA patients showed altered glucose metabolism in RSA patients.^[30]^ Therefore, the role of LM in RSA is multifaceted, involving glycolysis in trophoblastic cells, polarization of macrophages, and glucose metabolism in the uterine microenvironment. In the present study, 2 LM-RGs, *CALR* and *PIEZO1*, were identified as key genes involved in RSA, providing an important theoretical basis for further exploration of the pathogenesis of RSA.

The *CALR* gene, which encodes calreticulin protein, ensures the correct folding of newly formed proteins and participates in maintaining the correct level of calcium ions in the endoplasmic reticulum.^[31,32]^ Currently, there is no direct evidence linking the *CALR* gene to RSA. However, research on related genes and mechanisms suggests that the *CALR* gene, which is associated with calcium homeostasis and apoptosis, may be linked to susceptibility to RSA.^[31]^ Abnormalities in the immune system may be one of the key factors leading to RSA.^[33]^ The *CALR* gene could affect the immune system and thereby contribute to RSA.^[33]^ The *CALR* gene could regulate the expression or function of cytokines whose polymorphisms are associated with the risk of RSA.^[34]^ The *CALR* gene may also affect embryo development and implantation by interacting with lncRNAs.^[35]^ In a report, it was suggested that a history of previous miscarriages can predict subsequent miscarriages, while treatment with aspirin during pregnancy or the presence of *CALR* mutations was associated with a lower risk of miscarriage.^[36]^ Another similar study showed that the benefits of aspirin treatment in preventing early pregnancy miscarriage were significant in JAK2 mutation cases and *CALR* mutation cases, and *CALR* mutation can predict maternal bleeding.^[32]^ In the process of LM, the *CALR* gene may influence lactate production and utilization by regulating the expression of related genes.^[31]^ Future studies can further explore the specific mechanisms by which the *CALR* gene influences RSA.

The *PIEZO1* gene, as a mechanically sensitive ion channel, converts mechanical forces into biological signals and plays a vital role in diverse BPs.^[37]^ In recent years, studies have found that *PIEZO1* has significant functions in different types of cells and tissues, possibly by regulating purine metabolism (especially GUK1, POLD1, and APRT).^[38]^ Research has found that *PIEZO1* is present in human fetal placental endothelial cells and has functional activity.^[39]^ The destruction of *PIEZO1* can prevent the normal response to shear stress.^[40]^ In addition, *PIEZO1* has been identified as a mechanosensitive cation channel that is downregulated in the uterine smooth muscle and microvascular endothelium of the uterine muscle layer during preterm birth.^[41]^ Although the specific role of the *PIEZO1* gene in RSA is not yet clear, it can be inferred that *PIEZO1* may influence embryo implantation and early development by affecting cellular mechanical sensing and signal transduction. Therefore, future research could further explore the potential role of the *PIEZO1* gene in RSA.

To translate the identification of *CALR* and *PIEZO1* as key genes into clinical practice, several approaches for detecting their mutations or altered expression could be considered. For mutation screening, targeted next-generation sequencing panels or Sanger sequencing of specific exons could be employed to identify known or novel variants in these genes in patients with RSA. For assessing gene expression levels, which was the focus of our study, RT-qPCR of endometrial biopsy samples, as we performed, is a standard and reliable method. Furthermore, immunohistochemistry could be utilized to evaluate CALR and PIEZO1 protein expression and localization in tissue sections. The choice of test would depend on the clinical context.

In the pathological mechanisms of RSA, the expression patterns of cytokines and their receptors undergo significant changes.^[42]^ For example, studies have found that in the trophoblastic tissue of patients with RSA, the expression of TGF-β3 and IL-25 was significantly downregulated, while the expression of IL-2RA was significantly upregulated.^[43]^ Cytokine gene polymorphisms are associated with the risk of RSA. Among them, the IL1B-511 T>C and IL6-634C>G gene polymorphisms are significantly linked to the RSA risk. The former may be a crucial risk factor, while the latter may have a protective effect.^[34]^ As evidenced by transcriptomic analysis of placental RNA expression microarrays or RNA sequencing of miscarriages (including spontaneous miscarriage, recurrent miscarriage, and stillbirth) and spontaneous preterm birth, the cytokine–cytokine receptor interaction pathway is a common pathway for premature birth and miscarriage.^[44]^ In obesity-related osteoarthritis, there is a close link between LM and cytokine production.^[45]^ Studies have shown that saturated fatty acids, such as stearic acid, can promote lactate production, thereby increasing the expression of VEGF and pro-inflammatory cytokines.^[45]^ This study showed that the pivotal genes related to LM in RSA are predominantly expressed within the cytokine–cytokine receptor interaction pathway. GSVA showed that certain aspects of cytokine–cytokine receptor interactions are suppressed in this disorder. These findings collectively reveal the potential mechanism of key genes in RSA and clarify the correlation between these genes and disease development.

In terms of treatment, the options for RSA are relatively limited, mainly due to the diversity and complexity of its causes.^[46]^ Current treatment strategies typically include personalized therapies targeting known etiologies, such as hormone replacement therapy, immunotherapy, and anticoagulation therapy.^[47]^ However, since the exact cause of RSA remains unclear, the effectiveness of many treatments still needs further validation.^[48]^ It was found that the *CALR* regulatory network is more complex than that of *PIEZO1*, and the 2 genes have common upstream regulators and share certain targeted drugs, such as estradiol and cyclosporine. Modulation of estrogen (e.g., estradiol) and progesterone levels in patients with RSA has been reported to improve the pregnancy success rate in patients.^[49]^ Cyclosporine, by suppressing excessive immune responses as an immunosuppressant and potentially helping to prevent maternal immune rejection of the fetus, is also used in the treatment of RSA.^[49]^ Therefore, our study provides a basis for targeted regulation of key genes for the treatment of RSA. Through personalized hormone adjustment and immune regulation, it may effectively improve the success rate of pregnancy and help patients achieve the goal of healthy pregnancy.

This study identified 2 key genes, *CALR* and *PIEZO1*, through bioinformatics methods. Furthermore, GSEA and other analyses were performed to explore their potential roles in RSA, providing new insights into targeted therapy for RSA. However, our research was mainly performed based on existing datasets, and further clinical trials are needed to validate our results. In addition, we will continue to investigate the roles of these mechanisms, reflecting the extension and sustainability of this study.

## Acknowledgments

We would like to express our sincere gratitude to all individuals and organizations who supported and assisted us throughout this research. Special thanks to the following authors: Daili Tang, Ting Lai, Lin Chen, Huiling Zheng, and Zhi Huang. We confirm that all named individuals (Daili Tang, Ting Lai, Lin Chen, Huiling Zheng, and Zhi Huang) have agreed to be acknowledged in the manuscript. This work was financially supported by the Science and Technology Plan Project of Guiyang City (Grant No. 2022-4-11-3).

## Author contributions

**Data curation:** Daili Tang, Huiling Zheng, Zhi Huang.

**Funding acquisition:** Ting Lai.

**Investigation:** Lin Chen.

**Project administration:** Daili Tang, Ting Lai.

**Resources:** Lin Chen.

**Supervision:** Daili Tang.

**Validation:** Huiling Zheng, Zhi Huang.

**Writing – original draft:** Daili Tang.

**Writing – review & editing:** Ting Lai.












